# High burden of infections caused by ESBL-producing MDR *Escherichia coli* in paediatric patients, Yangon, Myanmar

**DOI:** 10.1093/jacamr/dlab011

**Published:** 2021-02-14

**Authors:** Thida San, Ingyin Moe, Elizabeth A Ashley, Nilar San

**Affiliations:** 1 Yangon Children’s Hospital, Yangon, Myanmar; 2 Myanmar Oxford Clinical Research Unit, Yangon, Myanmar; 3 Lao-Oxford-Mahosot Hospital-Wellcome Trust Research Unit, Vientiane, Lao People's Democratic Republic; 4 Centre for Tropical Medicine and Global Health, University of Oxford, Oxford, UK; 5 University of Medicine 2, Yangon, Myanmar

## Abstract

**Background:**

There is mounting evidence of a high burden of antimicrobial-resistant infections in children in low- and middle-income countries (LMICs).

**Objectives:**

To detect the frequency of ESBL-producing *Escherichia coli* in clinical specimens from paediatric patients attending Yangon Children’s Hospital in Myanmar.

**Methods:**

All children attending Yangon Children’s Hospital who had clinical specimens submitted to the hospital diagnostic microbiology laboratory from June 2019 to December 2019 were included in the study. Specimens were processed routinely using standard methods with BD Phoenix used for pathogen identification and susceptibility testing. Presence of ESBLs was determined using the cephalosporin/clavulanate combination disc method with confirmation by PCR.

**Results:**

From 3462 specimens submitted to the Microbiology Laboratory, a total of 123 *E. coli* were isolated. Among them, 100 isolates were phenotypically ESBL producers, 94 (76.4%) of which were confirmed by PCR [82/94 (87%) CTX-M, 72/94 (77%) TEM, 1/94 (1%) SHV]. Most of the ESBL-producing *E. coli* were isolated from urine samples (52.1%, 49/94) and the majority were from the surgical unit (61.7%, 58/94). Only 34/94 (36%) isolates were susceptible to meropenem.

**Conclusions:**

This study confirms a high proportion of infections caused by ESBL-producing and MDR *E. coli* in children hospitalized in Yangon, where access to effective second-line antimicrobials is limited.

## Introduction

The majority of the growing evidence base for the high burden of antimicrobial resistance (AMR) in low- and middle-income countries (LMICs) comes from surveillance of the adult population. However recent meta-analyses focused on paediatric and neonatal patients in sub-Saharan Africa have shown that antimicrobial resistance rates are also increasing in this group, particularly in the Enterobacterales order, in which the rising rates of ESBL production are a major problem.^1^^,^^2^ ESBLs are classified into nine distinct structural/evolutionary families based on their amino acid sequences. The molecular epidemiology of ESBLs in Enterobacterales changed dramatically in the late 1990s with the recognition of community and hospital-acquired CTX-M-type ESBLs,^3^ named for their activity against cefotaxime. CTX-M β-lactamases have spread rapidly over the last two decades to become the predominant ESBL in many regions.^4^

Southeast Asia is thought to have one of the highest risks of AMR in the world, related to population density, high burden of disease and widespread availability of antibiotics.^5^ An analysis of 10 years of microbiological culture data from blood or other normally sterile site specimens taken from children hospitalized in Cambodia between 2007 and 2016 found AMR rates were high, particularly in Gram-negative organisms. Resistance to third-generation cephalosporins was detected in 78.8% (115/146) of *Klebsiella pneumoniae* isolates, and 49.5% (53/107) of *Escherichia coli* isolates.^6^ Recent data from the global Neonatal AMR research network (NeoAMR), established in September 2017, which includes neonatal units in India, China, Thailand, Bangladesh and Cambodia, showed high rates of resistance to third-generation cephalosporins in Gram-negative bacterial isolates.^7^

Myanmar shares borders with China, Laos, Thailand, India and Bangladesh. A retrospective analysis of blood culture data from Yangon General Hospital, a tertiary hospital for adults, between 2005 to 2013 reported 37/45 *E. coli* isolates as resistant to ceftriaxone; however confirmatory testing for ESBLs was not performed.^8^ Another study of bloodstream infections in patients in three hospitals in Yangon conducted between July and December 2014 confirmed 16/42 (38%) Gram-negative isolates were ESBL-producing Enterobacterales and 6/42 (14%) were New Delhi metallo-beta lactamase (NDM) carbapenemase producers.^9^ In another tertiary hospital in the north of Yangon in 2016, almost half (49.1%) of all *E. coli* isolates were ESBL producers.^10^ There are no data published on the prevalence of ESBL-producing Enterobacterales in the Myanmar paediatric population.

In this cross-sectional study we aimed to detect the proportion of ESBL-producing *E. coli* from all children attending Yangon Children’s Hospital who had clinical specimens submitted to the hospital diagnostic microbiology laboratory from June 2019 to December 2019. Yangon Children’s Hospital was established in 1970 and is the national referral hospital for paediatrics in Myanmar. This 550 bed primary to tertiary hospital admits 25 000–30 000 children per year, and there are approximately 90 000–100 000 outpatient consultations.

## Patients and methods

All sequential clinical specimens from paediatric patients (blood, sputum, urine, CSF, fluid and pus/wound swabs) submitted to the Microbiology Laboratory of Yangon Children’s Hospital for routine culture and susceptibility testing were included in the study. Routine processing (microscopy, culture and identification, phenotypic ESBL detection) was performed. Identification and antibiotic susceptibility testing were performed using the BD Phoenix (Becton Dickinson Automated Microbiology System). Full details of laboratory methods and compliance with the Microbiology Investigation Criteria for Reporting Objectively (MICRO) checklist^11^ are in the Supplementary data (available at *JAC-AMR* Online).

*E. coli* isolates resistant to third-generation cephalosporins (ceftriaxone, ceftazidime or cefotaxime) (MIC ≥2 mg/L) were suspected of being ESBL producers, in accordance with CLSI guidance (2019).^12^ ESBL screening test-positive *E. coli* isolates were phenotypically confirmed by the cephalosporin/clavulanate combination disc method. ESBL genes (CTX-M, TEM and SHV) were detected by multiplex PCR (see Tables S1 to S3 and Figure S1 for methods and primers). Data analysis was done using SPSS software version 16.

## Results

Between June 2019 and December 2019, 3462 clinical specimens (1637 blood cultures) were submitted to the Microbiology Laboratory of Yangon Children’s Hospital for routine culture and susceptibility testing with no duplicates. A total of 123 *E. coli* were isolated (10 from blood cultures), of which 100 were resistant to third-generation cephalosporins, and 94 (76.4%) were confirmed as ESBL producers by PCR. Of the 94 children with confirmed ESBL, 55 (59%) were male and the median (range) age was 2 years (5 days to 12 years). Six specimens came from neonates. The specimen types were urine (*n = *49; 52%;), wound swabs (*n = *32; 34%), blood (*n = *6; 6%), pus (*n = *3; 3%), fluids (2; 2%), sputum (*n = *1; 1%) and an intravenous catheter tip (*n = *1; 1%). Among the 49 urine specimens, 31 were clean-catch specimens, 1 was obtained by suprapubic aspiration, 15 were catheter specimens, and 2 were from nephrostomies. Among the 32 wound swabs and 3 pus specimens, the majority (*n = *28) were from post-operative wounds after abdominal surgery.

Most ESBL-producing *E. coli* were isolated from specimens sent from the surgical unit (*n = *58; 62%), followed by general medical wards (*n = *21; 22%) and then haematology–oncology (*n = *6; 6%) with only two specimens originating from the neonatal unit (see full list in Table S4).

Among the 94 ESBL-producing *E. coli* isolates, 82 (87.2%, 82/94) were CTX-M gene positive, 72 (76.6%, 72/94) were TEM gene positive, and only 1 (1.1%, 1/94) was SHV gene positive. Several isolates were positive for more than one gene (Table 1).

**Table 1.** Frequency of different types of ESBL genes among *E. coli* isolates

ESBL gene	Invasive isolates (*n*)^a^	Non-invasive isolates (*n*)	Total, *n* (%)
CTX-M only	1	21	22 (23)
TEM only	1	11	12 (13)
Both CTX-M + TEM	7	52	59 (63)
All CTX-M + TEM +SHV	0	1	1 (1)
Total	9	85	94

a Invasive isolates: blood (6), peritoneal fluid (1), pleural fluid (1), femoral catheter tip (1).

The antibiotic susceptibility patterns of ESBL-producing *E. coli* isolates are shown in Table 2. A very high proportion of isolates were resistant to carbapenems (62%), gentamicin (71%) or amikacin (47%). For the 59 isolates resistant to meropenem, MICs were >8 mg/L in 29 cases, and 4–8 mg/L in 30 cases.

**Table 2.** Antibiotic susceptibility^a^ patterns of isolated ESBL-producing *E. coli*

Antibiotic	Susceptible, *n* (%)	Intermediate, *n* (%)	Resistant, *n* (%)
Amikacin	49 (52)	1 (1)	44 (47)
Amoxicillin/clavulanic acid	4 (4)	5 (5)	85 (90)
Cefotaxime	0	0	94 (100)
Ceftriaxone	0	0	94 (100)
Ceftazidime	4 (4)	4 (4)	86 (91)
Cefepime	0	7 (7)	87 (93)
Ciprofloxacin	18 (19)	1 (1)	75 (80)
Colistin	94 (100)	0	0
Gentamicin	27 (29)	0	67 (71)
Imipenem	32 (34)	6 (6)	56 (60)
Meropenem	34 (36)	1 (1)	59 (62)
Piperacillin/tazobactam	25 (27)	4 (4)	65 (69)
Trimethoprim/sulfamethoxazole	8 (9)	40 (43)	46 (49)

a CLSI guidance (2019)^12^ was used to define susceptibility.

Among 38 *E. coli* isolates from urine specimens tested against nitrofurantoin, 22 (58%) were susceptible (MIC ≤32 mg/L). Fosfomycin MIC results from BD Phoenix for urine isolates are shown in Table S5.

## Discussion

The global increase in antimicrobial resistance is a major concern as patient outcomes are compromised and it increases the financial burden on healthcare systems. The emergence of ESBL-mediated antimicrobial resistance in Gram-negative bacteria is a growing threat, with CTX-M predominating.^4^^,^^13^*E. coli* is the most common cause of community- and hospital-acquired bacterial infections. ESBL-producing strains frequently harbour co-resistance genes conferring resistance to other antimicrobial agents. Our results suggest rates of ESBL-producing *E. coli* are higher in hospitalized children than adults in Yangon; however, this may reflect sampling bias. In addition, we found a very high burden of carbapenem resistance (62% of isolates) which presents major challenges in treating children with serious infections. The first line treatments for sepsis in Myanmar are ampicillin plus gentamicin for neonates and ceftriaxone for older children. Access to second-line antibiotics is limited. Amikacin, tigecycline and colistin are available in the country, however colistin is rarely used in children. Most of the isolates in our study were from urine specimens for which the first line treatment is oral amoxicillin or co-amoxiclav or cefalexin. Tigecycline is not usually recommended for the treatment of urinary tract infections because low concentrations are achieved, however there are reports of successful outcomes.^14^ None of the isolates in this study was resistant to colistin, however the *mcr-1* gene was detected in *E. coli* causing a urinary tract infection in a patient in Yangon in 2018.^15^ Nitrofurantoin and fosfomycin could be options for the treatment of uncomplicated urinary tract infection for some children in the future.

Limitations of this analysis include lack of clinical outcome data and our inability to verify carbapenem resistance mechanisms in this sample, or to distinguish community-acquired from hospital-acquired infections. However, regardless of this, the results reveal a very high burden of antimicrobial-resistant infections, which presents major challenges for the treatment of hospitalized children in Yangon.

## Supplementary Material

dlab011_Supplementary_Data
