# Supplementary material for: High burden of infections caused by ESBL-producing MDR Escherichia coli in paediatric patients, Yangon, Myanmar
Source: JAC Antimicrob Resist. 2021 Feb 14;3(1):dlab011. doi: 10.1093/jacamr/dlab011 (PMC7882151; doi:10.1093/jacamr/dlab011)
Supplement: dlab011_Supplementary_Data [file dlab011_supplementary_data.docx]

**Supplementary data**

**Contents**

**A Laboratory Methods**

**B Supplementary results**

**C MICRO checklist**

**A Laboratory methods**

Laboratory work was done in the Microbiology Laboratory of Yangon Children’s Hospital. The laboratory is not accredited through a national or international body. The laboratory participates to the national External Quality Assurance scheme organized by the National Health Laboratory, Yangon, Myanmar.

1. **Identification, isolation and antimicrobial susceptibility testing of *E. coli***

**Microscopic examination of the specimen after Gram staining**

After inoculation onto blood agar and MacConkey agar, specimen was smeared on a clean glass slide, heat fixed and stained with Gram's stain. The stained smear was examined under the oil immersion lens (1000X) of ordinary light microscope.

**Isolation of *Escherichia coli* by culture**

**(a)** **Culture**

Each specimen was inoculated on sheep blood agar and MacConkey agar. Blood agar plates for sputum and wound swab samples were incubated at 37°C with 3-5% CO_2_ overnight.

*E.coli* was first identified by colonial morphology. *E.coli* can be found circular, convex, grey, translucent colonies on Blood agar. On MacConkey agar, they produce deep red colonies as the organisms are lactose fermenters.

**(b) Gram’s stain of suspected colonies**

A likely colony from a culture plate was taken with a wire loop and suspended in a loopful of normal saline on a clean glass slide and heat fixed. Smears were stained with Gram’s stain and examined under oil immersion lens of the ordinary light microscope.

Identification and Antibiotic susceptibility testing

Identification and antibiotic susceptibility testing of *E.coli* was performed by Automated Microbiology System (BD Phoenix Becton Dickinson).

**Detection of ESBL isolates by phenotypic tests**

The isolates, resistant to ceftriaxone, ceftazidime and cefotaxime were suspected of ESBLs producer and tested of ESBL production by phenotypic confirmatory tests.

**Initial screening test for ESBL**

The breakpoints of minimum inhibitory concentration (MIC) ≥2 µg/ml of ceftazidime, ceftriaxone and cefotaxime were indicative of suspicion for ESBL production according to the CLSI, (2019) guideline.^1^

**Phenotypic confirmatory tests for ESBL production**

In this study, initial ESBL screening test positive *E.coli* isolates were phenotypically confirmed by the cephalosporin/clavulanate combination disc method.

Discs containing ceftazidime, ceftazidime plus clavulanic acid and cefotaxime, cefotaxime plus clavulanic acid were placed on Mueller Hinton agar center to center at least 25 mm apart. After 16-18 hours incubation at 37ºC, ≥ 5mm increased in a zone diameter for either antimicrobial agent tested in combination with clavulanic versus its zone when tested alone were considered positive for ESBL production.^1^

1. **Detection of ESBL genes (CTX-M, TEM** **and** **SHV) by multiplex-PCR**

**DNA Extraction from colony**

DNA extraction was performed according to nucleic acid testing and sequencing work guideline, Department of Hygiene, Sapporo Medical University School of Medicine, Sapporo, Japan. 100μl of TNE buffer (10mM Tris-HCl, 0.1M NaCl, 1mM EDTA, pH 7.5) was dispensed into 1.5ml-tube. The bacterial growth from agar plate was taken and dissolved in TNE in the 1.5ml tube and then centrifuged at (10000rpm for 1min). After that 100μl of NaOH (50mM) was added and mixed by vortexing. The suspension was heated at 40º C for 10 min. Then 16μl of 1M Tris-HCl (pH6.8) was added and mixed. After centrifugation at 10,000 rpm for 1min, the supernatant was transferred into 1.5 ml eppendorf tube and １μl of supernatant was used as the DNA sample for PCR.

**Master Mix (or) Premix PCR mixture preparation**

The reaction mixture was prepared as shown in Supplementary table (ST 1).

**Table S1 PCR Reaction Mixture (Master Mix)**

| **Components** | **Volume (μl) / Reaction** |
| --- | --- |
| Sterile double distilled water | 16.5 |
| 10X Ex Taq buffer | 5 |
| dNTP (2.5mM) | 2 |
| Primer mixture | 0.5 |
| Takara Ex Taq DNA polymerase (5units/μl) | 0.5 |
| DNA sample | 1 |
| **Total** | 25.5 |

Dispense 24.5μl of master mix into 20 μl PCR tube, and 1μl of DNA sample was added to each reaction mixture tube. Mixed the reaction mixture by vortexing.

**Procedures of amplification by PCR**

All DNA samples were tested for the genes encoding TEM, SHV and CTX-M β-lactamases by multiplex PCR assay using the primers listed in Table S2. The samples were amplified in a DNA thermal cycler as shown in Table S3.

**Table S2 Primers for detection of β-lactamase genes ^2^**

| **β-lactamase genes** | **Primer sequence (5’ to 3’)** | **PCR Product (bp)** |
| --- | --- | --- |
| bla-SHV-. F | ATGCGTTATATTCGCCTGTG | 747 |
| bla-SHV- R | TGCTTTGTTATTCGGGCCAA | 747 |
| bla-TEM-F | TCGCCGCATACACTATTCTCAGAATGA | 445 |
| bla-TEM-R | ACGCTCACCGGCTCCAGATTTAT | 445 |
| bla-CTX-M-F | ATGTGCAGYACCAGTAARGTKATGGC | 593 |
| bla-CTX-M-R | TGGGTRAARTARGTSACCAGAAYCAGCGG | 593 |

F = Forward primer R = Reverse primer

**Table S3 Thermal cycling condition for PCR reaction**

| **Step** | **Temperature (°C)** | **Time** | **No. of cycles** |
| --- | --- | --- | --- |
| Initial denaturation | 94 | 1 min | 1 |
| Denaturation | 94 | 30 sec | 30 |
| Annealing | 55 | 30 sec |  |
| Extension | 72 | 1 min |  |
| Final extension | 72 | 5 min | 1 |

**Detection of PCR product by Agarose gel electrophoresis**

PCR products were detected by electrophoresis on 2% agarose gel in 1X TAE buffer containing ethidium bromide (1μg/ml) at 100V for 30min. Presence of amplified DNA and its size was observed by UV transilluminator and photographed.


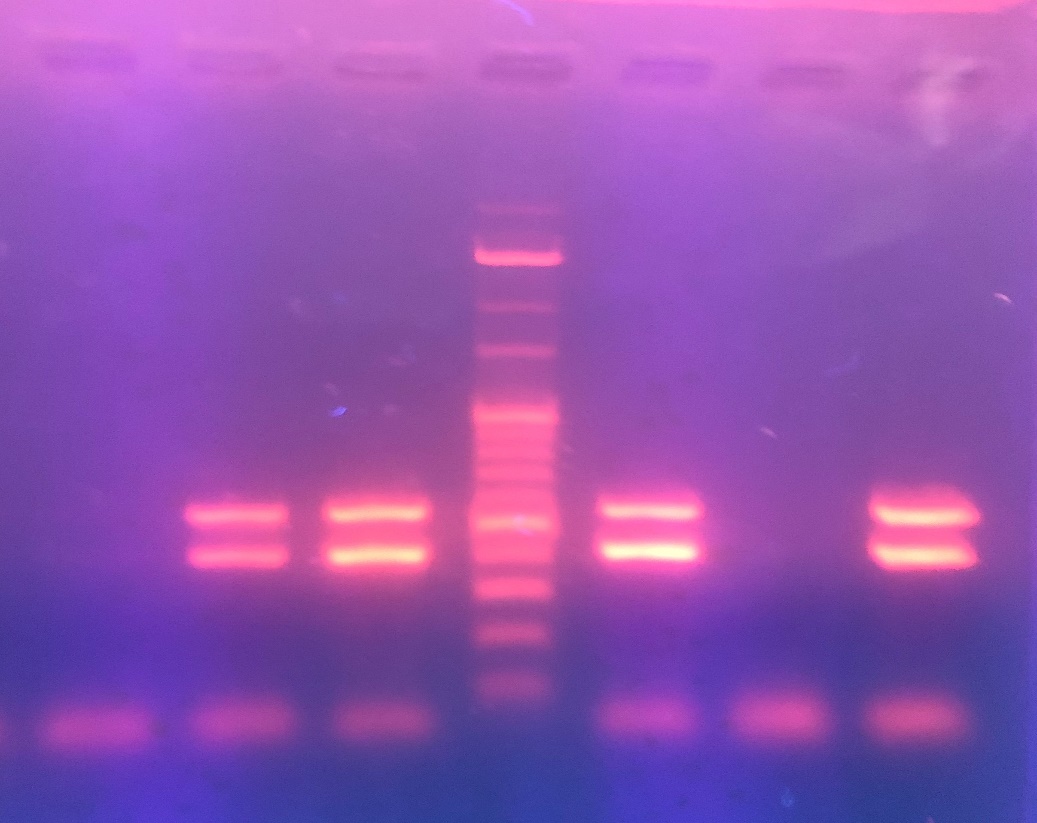


Lane-1 Lane-2 Lane-3 Lane-4 Lane-5 Lane-6 Lane-7

TEM 445 bp

CTX-M 593 bp

**Figure S1** **Detection of ESBL genes by Multiplex PCR**

Lane-2, lane-3, lane-5, lane-7: CTX-M and TEM positive

Lane-1, lane-6: ESBL gene negative

Lane-4: DNA ladder (100bp)

**B Supplementary results**

**Table S4. Distribution of ESBL producing *E coli* among different clinical inpatient departments**

| Department | **ESBL producing *E coli***  N(%) |
| --- | --- |
| Surgical | 58 (62) |
| Medical | 21 (22) |
| Haematology- Oncology | 6 (6) |
| ICU | 4 (4) |
| Neurology | 2 (2) |
| Neonatal | 2 (2) |
| Renal | 1 (1) |
| Total | 94 |

**Table S5 Fosfomycin MIC results (BD-Phoenix) for isolates from urine**

| Interpretation | MIC mg/L | N |
| --- | --- | --- |
| Sensitive | <= 16 | 33 |
|  | 32 | 1 |
|  | 64 | 1 |
| Resistant | >64 | 1 |
| Not tested | | 13 |
| Total | | 49 |

Note CLSI (2019) only recommends agar dilution to assess fosfomycin susceptibility

C. **MICRO Checklist^3^**

| Item | Number | Recommendation | Completed Y/N | |
| --- | --- | --- | --- | --- |
| Methods |  |  |  | |
| *Study design* | 1* | **Specimen types**: Describe the types of specimen included, i.e. clinical (e.g. blood cultures) or non-diagnostic surveillance (e.g. admission and other screening swabs to diagnose carriage). If specimens were obtained for diagnostic reasons, clinical syndromes should be described where possible, and specimens / isolates stratified by clinical syndrome. | Y (syndromes not available) | |
|  | 2* | **Sampling period:** State the collection timeframe for specimens yielding isolates for which data is reported, e.g. from MM/YY to MM/YY to be able to identify variability between seasons. | Y | |
|  | 3* | **Sampling strategy:** Describe the strategy for specimen collection, e.g. asymptomatic screening, sampling of all febrile patients, sampling at clinician discretion, sampling of specific patient groups, convenience sampling (e.g. use of isolates from an existing sample repository). Specify whether sampling followed routine clinical practice or was protocol driven. Classify specimens as from community-acquired (CAI) or hospital-acquired (HAI) infections. The definition of HAI used (e.g. HAI defined by specimen collection >48h after hospital admission) should be provided and should use ideally an international standard (e.g. US-Centers for Disease Control). | Y; CAI/HAI distinction not possible |  |
|  | 4 | **Target organisms:** Explicitly state which organisms / organism groups were included in the report. Nomenclature should follow international standards (i.e. using approved genus / species names as summarised in the International Journal of Systematic and Evolutionary Microbiology). Lists of approved bacterial names can be downloaded from Prokaryotic Nomenclature Up-to-Date (<https://www.dsmz.de/bacterial-diversity/prokaryotic-nomenclature-up-to-date.html>) and the List of Prokaryotic Names with Standing in Nomenclature (<http://www.bacterio.net/>). Organisms considered contaminants should be listed, if appropriate (e.g. coagulase negative staphylococci or *Corynebacterium* spp.. | Y |  |
| *Setting* | 5* | **Geographical setting:** Describe the geographical distribution of specimens / patients from which isolates were obtained; at least to a country level, but preferably to a sub-national level or a geoposition. | Not available | |
|  | 6* | **Clinical setting:** Describe the type and level of the healthcare facilities (e.g. primary, secondary, tertiary) from which specimens were obtained. If stating a microbiology laboratory, the centres served by the laboratory should be specified. | Y | |
| *Laboratory work* | 7 | **Specimen processing:** If applicable, describe specimen collection and handling, processing and sub-culture methods for all types of specimen included. For example, if reporting AST results for blood culture and cerebrospinal fluid culture isolates, the processing of these specimens by the laboratory should be briefly explained, including how specimens are sub-cultured, the media used, incubation conditions and duration. A summary of specimen processing steps (e.g. pre-processing steps, nucleic acid extraction method (if applicable), amplification platform, contamination avoidance strategy) should be provided for molecular-only workflows (e.g. to detect *Mycobacterium tuberculosis* and rifampicin resistance using the Cepheid Xpert MTB/RIF system). | Y (in supplementary material) | |
|  | 8* | **Target organism identification:** Details of identification methodology should be reported briefly. Where identification databases were used (e.g. bioMerieux API / bioMerieux VITEK-MS / Bruker Biotyper), the version should be specified.  In general, all pathogens should be identified to species level. In the case of *Salmonella* species, organisms should be identified to at least the *S.* Typhi, *S.* Paratyphi, or non-typhoidal salmonella (NTS) level. Strain subtyping methods should be reported according to STROME-ID. | Y |  |
|  | 9* | **Antimicrobial susceptibility testing:** Describe the antimicrobial susceptibility testing methods used, internal quality control processes, and their interpretation, with reference to a recognised international standard – e.g. CLSI, EUCAST. Where an international standard was followed, the specific edition(s) of guidelines used should be referenced. Deviations from standard methodology should be described, along with evidence of validation. Handling of any changes to interpretative criteria during the sampling period should be documented. State whether the raw AST data (zone diameters and / or minimum inhibitory concentrations) were re-categorised with updated breakpoints or left as-is. | Y (CLSI 2019) | |
|  | 10 | **Additional tests performed to identify resistance mechanisms:** Describe the testing methods used for adjunctive / confirmatory antimicrobial susceptibility tests, such as enzymatic / molecular assays (e.g. Xpert MTB/RIF, mecA PCR) and inducible resistance assays, with reference to a recognised international standard, where available. Where an international standard was followed, the specific edition of guidelines used should be referenced. Deviations from standard methodology should be described, along with evidence of validation. | Y |  |
|  | 11* | **Antimicrobial resistance definitions:** Define resistance for each antimicrobial class (i.e. are isolates in the “intermediate” category included within “susceptible” or “resistant” or analysed as a distinct category). If using the term, define MDR (e.g. ≥1 agent in ≥3 classes tested). For each organism type, an MDR test panel must be defined, consisting of the minimum panel of individual antimicrobial agents / classes against which an isolate must be tested for that isolate to be considered tested for MDR status. Antimicrobials to which an organism is intrinsically resistant cannot be part of the test panel or contribute to MDR status. | Y |  |
| *Quality assurance* | 12* | **External quality assurance:** State whether the microbiology laboratory participates in an external quality control programme and, if so, provide scheme details. Examples include the UK National External Quality Assurance Scheme ([www.ukneqasmicro.org.uk](http://www.ukneqasmicro.org.uk)) and the American College of Pathologists External Quality Assurance / Proficiency Testing Program (<http://www.cap.org/web/home/lab/international-laboratories/external-quality-assurance-proficiency-testing-international-laboratories>) | Y |  |
|  | 13 | **Accreditation:** State whether the laboratory is accredited through a national or international body (e.g. the International Standards Organisation, ISO) and specify which assays are covered in the accreditation. | Y (no accreditation) | |
| *Bias* | 14* | **Duplicate and sequential isolates:** The strategy for accounting for duplicate and sequential isolates from the same patient should be clearly detailed. Duplicate isolates are multiple isolates of the same phenotypic organism (i.e. same species and same resistance profile) from the same patient on the same date cultured either from the same clinical specimen, or from two separate clinical specimens, such as blood and CSF. Sequential isolates are isolates of the same phenotypic organism from the same patient at different dates, such as blood cultures taken on different dates. Various strategies for the handling of duplicate and sequential isolates exist, and the strategy used should be transparent as it will bias pooled resistance results. For example, inclusion of all isolates (the ‘all isolate strategy’), has been shown to shift pooled resistance proportions toward greater resistance, whilst inclusion of only the first isolate per patient (the ‘first isolate strategy’) or only the first isolate per infection episode (the ‘episode-based strategy’) will shift pooled results towards susceptibility. | Y |  |
| Results |  |  |  |  |
|  | 15* | **Population:** Describe the demographics of the population from which clinical specimens and subsequent isolates have been obtained, disaggregating age and gender data. | Y | |
|  | 16* | **Denominators:** Patient and isolate denominators should be used appropriately to ensure clarity regarding the numbers included in each analysis. Of particular importance is the reporting of resistance where first- and second-line AST panels were used (i.e. not all isolates of a particular species were tested against all agents). For drugs where only a subset of isolates were tested, reporting of a percentage without the numbers of isolates tested / resistant may be highly misleading. | Y | |
|  | 17 | **Site / place of acquisition**: AST data from CAI and HAI should be reported and analysed separately. | NA |  |
|  | 18 | **Reporting resistance proportions for single agent and class resistance:** Proportions of resistant isolates should be reported as number of isolates susceptible or resistant to a given antimicrobial agent / class out of actual number of isolates tested for susceptibility to that agent / class. | Y |  |
|  | 19 | **Reporting multidrug resistance proportions:** If defined, the proportion of MDR isolates should be expressed as the number of MDR isolates out of the number of isolates tested (i.e. the number undergoing the MDR test panel specific to that organism). Single agent / class resistance should be always be reported, regardless of MDR reporting. | Not defined |  |
| Discussion |  |  |  | |
| *Limitations* | 20 | Discuss any reasons why bias may have been introduced into the reported data, due to patient / specimen selection, isolation of organisms, or otherwise. Consider factors which may have either introduced bias into the types of organisms isolated or the antimicrobial susceptibility profiles, e.g. receipt of antimicrobials prior to specimen collection will reduce the yield of certain species and also select for more resistant organisms. | Y |  |

**References**

1. Clinical and Laboratory Standards Institute. Performance Standards for Antimicrobial Susceptibility Testing. 2019.

2. Lukac PJ, Bonomo RA, Logan LK. Extended-spectrum β-lactamase-producing Enterobacteriaceae in children: old foe, emerging threat. *Clin Infect Dis* 2015; **60**: 1389-97.

3. Turner P, Fox-Lewis A, Shrestha P *et al*. Microbiology Investigation Criteria for Reporting Objectively (MICRO): a framework for the reporting and interpretation of clinical microbiology data. *BMC Med* 2019; **17**: 70.
